# Readiness to Change: A Pathway to the Adoption of Trauma-Sensitive Teaching

**DOI:** 10.3390/bs12110445

**Published:** 2022-11-12

**Authors:** Megan A. Blanton, Fallon J. Richie, Jennifer Langhinrichsen-Rohling

**Affiliations:** 1 Department of Psychology, University of South Alabama, Mobile, AL 36688, USA; 2 Department of Psychological Science, University of North Carolina at Charlotte, Charlotte, NC 28223, USA

**Keywords:** trauma-sensitive schools, readiness to change, attitudes related to trauma-informed care

## Abstract

Creating a trauma-sensitive classroom requires a shift in perspective from viewing a student’s problematic behavior as a function of poor character to considering it contextually. However, a trauma-sensitive perspective may be insufficient for school staff to implement trauma-sensitive practices. Theoretically, motivation, or readiness to change (R2C), is needed to adopt any new behavior. Therefore, the purpose of this study was to examine the role of R2C in the relation between attitudes related to trauma-informed care (ARTIC) and the adoption of trauma-sensitive practices in a school setting. The targeted elementary school primarily serves Black students (83%), living below the federal poverty line. All staff attended an in-service training about trauma-sensitive schools (TSS), in which trauma-sensitive strategies were modeled, and student-friendly, emotional regulation materials were provided. Teachers and staff (*n* = 40) were assessed one year after receiving the TSS training. Participants reported their ARTIC, R2C, and trauma-informed strategy adoption. Using PROCESS Model 4, R2C fully mediated the relation between ARTIC and reported use of specific trauma-sensitive classroom strategies (β = 0.19, bootstrapped SE = 0.12, 95% LLCI = 0.04, 95% ULCI = 0.49). Facilitating R2C is essential when implementing trauma-sensitive school strategies. System-wide policies that may help promote the uptake of trauma-sensitive practices are described.

## 1. Introduction

A child’s development is highly dependent on contextual factors, including immediate family and home life, neighborhood, and school, as well as educational policies and culture at-large [1]. In a well-known study of adverse childhood experiences (ACEs) such as parental divorce, abuse or neglect, and parental substance use, Felitti and colleagues (1998) concluded that as many as half of U.S. adults have experienced at least one adverse or traumatic childhood event while a quarter will report two or more ACEs. Importantly, the number of ACEs reported was directly related to adult physical and mental health outcomes, including heart, lung, and liver disease as well as depression and suicide attempts [2]. Similar findings have been replicated recently with diverse samples [3,4]. Childhood trauma has also been associated with IQ deficits, learning and concentration impairment, and behavioral problems [5]. Thus, the impact of childhood trauma may be particularly salient to school adjustment and academic achievement [6]. As such, there has been a national push to create schools staffed by personnel who recognize the impacts of trauma and are sensitive and responsive to children who have experienced it. In particular, school personnel may need to be comfortable helping children regulate their emotions in order to prevent revictimization. Schools are positioned to serve as a safe-haven from what goes on at home (e.g., trauma) but until the early 2000s, there was an absence of research considering the importance of implementing trauma-informed or trauma-sensitive practices in schools. Teachers are essential to enacting trauma-sensitive classrooms in ways that will change school culture.

### 1.1. Adopting Trauma-Sensitive Practices in Schools

In 2014, the Substance Abuse and Mental Health Services Administration of the United States of America (SAMHSA) identified six key principles of trauma-informed care: creating a sense of safety; practicing trustworthiness and transparency; utilizing peer support; employing collaboration and mutuality; practicing empowerment and fostering voice and choice; and recognizing cultural, historical, and gender issues [7]. These principles have been suggested for a variety of settings including healthcare organizations, the justice system, and educational facilities [8]. Adoption of these trauma-informed principles is expected to create a school environment in which all children, including children with a trauma history, can meet their potential [5]. The first step teachers must take to effectively implement the core principles of trauma-informed care is to adopt a “trauma lens,” which includes viewing students’ behavior as rooted in context [9]. When teachers view students’ behavior in context, they ask themselves, “What happened to you?” rather than, “What’s wrong with you?” in response to inappropriate classroom behavior; this has been labelled the adoption of a “trauma lens”. Consistent with this perspective, numerous studies have established that increasing knowledge of trauma and receiving training in trauma-informed skills has a positive impact on teachers’ attitudes, preparedness, and self-efficacy about working with children with trauma histories or who are exhibiting difficult to manage classroom behavior [9,10,11]. However, no research to our knowledge has explored whether adoption of a “trauma lens,” is associated with the implementation of trauma-sensitive practices in classrooms. Though there is no universal agreement on the measurement of a trauma lens, the main instrument within the literature is the Attitudes Related to Trauma-Informed Care (ARTIC) scale [12]. Given that previous qualitative research has shown that teachers with more favorable attitudes towards trauma-informed care are more likely than teachers with less favorable attitudes to consider contextual factors (e.g., trauma history) as a potential source of student misbehavior [13], the ARTIC offers a quantitative measure of a “trauma lens.”

### 1.2. Readiness to Change

Theoretically, an important motivational component of adopting any new behavior is readiness to change. Readiness to change can occur at the individual or organizational level and indicates the extent to which individuals or organizations are likely to implement changes. In theory, readiness to change is made up of two components: change commitment and change efficacy [14]. People must both be confident and willing to commit to taking steps towards change and believe that the desired change is important. In clinical settings, simple 10-point rulers are frequently used to assess an individual’s readiness and confidence in their ability to change their behaviors as well as their perceptions of the level of importance of the new behavior [15]. These brief 10-point rulers have been demonstrated to perform equally, and in some cases better, than longer measures of change readiness [16].

Marvin and colleagues (2018) demonstrated that in a non-profit human service agency, employees’ favorable attitudes towards trauma-informed care were associated with higher readiness to change [17]. Interestingly, this study demonstrated that general knowledge about trauma-informed care was not necessarily associated with favorable attitudes related to trauma-informed care, nor was it associated with attitudes related to trauma-informed care. TIC trainings in school settings have also found that increasing a teacher’s knowledge of TIC is not inherently related to improving their attitudes towards TIC [10]. These findings indicate that knowledge alone may not be sufficient to develop a “trauma lens.” As such, it would be expected that simply providing teachers with increased knowledge of trauma-informed care, such as through educational offerings, might not be enough to ensure strategy implementation [17]. Instead, positive attitudes toward trauma-informed care would be expected to predict the adoption of new behaviors (i.e., implementation) primarily through the pathway of greater readiness to change. This model undergirds the current study.

Consistent with this premise, in a qualitative study of mental health and substance use service providers, provider and consumer readiness to change were identified as facilitators of trauma-informed practice implementation and delivery [18]. Thus, while previous studies have examined the association between attitudes related to trauma-informed care and readiness to change [17], as well as the association between readiness to change and adoption of trauma-informed practices [19], no identified studies have investigated the relation between trauma-informed attitudes and practice adoption, specifically as it is mediated by readiness to change. The present study fills this gap.

Further, while previous studies have addressed the impact of readiness to change on trauma-informed practice implementation outside of school settings (e.g., human service agencies, health care systems), we know considerably less about the impact of educator readiness to change on trauma-informed practice implementation. Importantly, there is also a lack of research on whether attitudes towards trauma-informed care directly predict strategy adoption in the classroom. It is possible that teachers or other service providers hold favorable attitudes towards trauma-informed care but are unable or unwilling to make the necessary changes to adopt the recommended practices. Thus, this paper will examine whether readiness to change plays an important role in the relationship between attitudes towards trauma-informed practices and adoption of trauma-informed strategies within an elementary school setting.

## 2. Materials and Methods

### 2.1. Setting

The present study was conducted in a Pre-K through 5th Grade elementary school located in an impoverished urban neighborhood within the southern United States. The elementary school had a history of collaboration with these authors (redacted for review) through an ongoing clinical service delivery project. Called the APPLE project (Attention Problems Present in the Learning Environment), an interdisciplinary clinical team accepted in-school referrals for school counseling, psychology, and psychiatry services. Many requests were for attention deficit disorder (ADD) evaluations for children displaying disruptive behaviors in the classroom. The clinical collaboration lasted for two years, during which time the clinicians noted that many of the children whom teachers referred for ADD evaluations did not meet criteria for ADD. Instead, many referred children had significant and untreated trauma histories. Thus, at the conclusion of the APPLE project, the clinical team conducted a professional development training for school administrators, teachers, and support staff aimed toward increasing awareness of the prevalence of trauma among at-risk children and its impact on students. The clinical team also modeled evidence-based trauma-sensitive behavioral management techniques and the team provided resources to continue these practices. The training will be discussed in further detail below. At the time of the training, this elementary school served 760 students, 83% of whom identified as Black or African American, and 81% of whom received free or reduced-price lunch [20].

### 2.2. Procedures

Clinicians from the APPLE team delivered the initial in-service training to all school faculty including school administrators, teachers, and support staff. The training concentrated on helping personnel realize, recognize, and respond to trauma expressed by children in their school while preventing revictimization. Specifically, participants learned about the definition, prevalence, and impact of Adverse Childhood Experiences (ACEs) on children’s school and learning behavior. School personnel also learned about the characteristics of trauma-informed schools including evidenced-informed behavior management techniques (e.g., labeled praise, active ignoring, direct brief commands, and cupcake breathing) [21,22], systems-level practices, and self-regulation and peer support strategies for teachers. The training included experiential practice activities with anonymous case examples of students with trauma histories. Following the training, all classrooms, the school counselor, and the library were provided with grade appropriate “coping boxes,” which were one of the trauma-sensitive practices taught in the training. Each coping box contained sensory toys, a timer, coloring sheets, and crayons. Teachers and the school counselor were shown how to utilize the materials within the coping box in follow-up, grade-level-tailored, demonstration sessions and in response to coaching requests. Finally, additional educational material about how to implement trauma-sensitive classrooms was gifted to the school counselor to facilitate her work with school personnel and to serve as a continued resource for the school.

The data used in the present study were generated in the third wave of data collection from a larger program evaluation study. The third wave of data was collected one year following competition of the initial TIC workshop described above. Data from this wave were selected for use in the present study, as it was the only wave of data collection that included the ARTIC scale. All surveys were completed with pen and paper.

### 2.3. Participants

Participants were school staff who participated in the initial training related to implementing trauma-sensitive classroom management strategies. Using an algorithm, each participant created and wrote a unique identifier at the top of the first page to maintain anonymity. This was also carried out so their attendance at the initial training could be verified (through the matching of the identifiers across each wave of data collection).

Of the 57 school-staff who participated in the initial training, 45 were available to complete the one-year follow-up survey and agreed to participate. Demographic data, such as job title and age, were gathered using broad categories to maintain confidentiality. All data were analyzed with the Statistical Package for the Social Sciences (SPSS) version 27.0 manufactured by International Business Machines (IBM; Armonk, NY, USA).

### 2.4. Measures

Attitudes Related to Trauma-informed Care. The Attitudes related to Trauma-Informed Care (ARTIC) ten-item scale [23] was used to measure participants’ trauma-informed lens. It is the only validated measure that assesses attitudes towards trauma-informed care. Thus, the ARTIC functioned as an assessment of each participant’s adoption of a “trauma lens.” Participants respond to each statement using a 7-point Likert scale ranging from trauma-informed care-favorable attitudes to trauma-informed care-unfavorable attitudes. For example, a trauma-informed care-unfavorable attitude is “students’ learning and behavior problems are rooted in their behavioral or mental health condition” and its opposite is “students’ learning and behavior problems are rooted in their history of difficult life events.” In the initial validation study, the ARTIC-10 produced good internal consistency (α = 0.82). In our sample, the internal consistency was adequate (α = 0.71).

Readiness to Change. Readiness to change was assessed via three questions that were derived from the transtheoretical model (TTM) and the motivational interviewing literature [24]. The three questions assessed staff’s perceptions of the importance, their confidence, and their readiness to implement the principles associated with a trauma-sensitive school. Participants rated each facet on a scale of 1 = strongly disagree to 10 = strongly agree. In this sample, the internal consistency of this 3-item scale was acceptable (α = 0.76).

Use of Trauma-Sensitive Practices. To assess the degree to which teachers adopted trauma-sensitive practices in their classrooms, self-report scales ranging from 1 = strongly disagree to 10 = strongly agree were utilized. Teachers rated the degree to which they used the five different strategies that had been explained, modeled, and promoted during the TIC training: direct, brief commands; active ignoring; labeled praise; coping boxes; and cupcake breathing. Responses to these items were averaged to create a composite skill use score. In this sample, this scale also demonstrated acceptable internal consistency (α = 0.78).

## 3. Results

### 3.1. Demographics

The final sample consisted of all school faculty and staff who were verified as having attended the initial trauma-sensitive schools training the previous year and who also completed the one-year follow-up assessment. Of the 45 who participated in wave three data collection, 40 completed all items on each of the measures used in analyses. Attendance at the trauma-sensitive school training was verified via receipt of the initial training baseline survey and/or self-reported attendance. Participants were generally between 35 and 44 years old (44.2%). The remaining participants identified as follows: 20.9% were between the ages of 25 and 34, 14% were between the ages of 45 and 54, 16.3% were between the ages of 55 and 64, and 4.7% of participants were between the ages of 65 and 74. Among the participants who agreed to report their sex, 92.1% were female and 7.9% were male; seven participants chose not to respond to this question. Of the participants who chose to report their race, 69.2% identified as Caucasian/White and 30.8% identified as African American/Black; six individuals preferred not to respond. The majority of the participants (86%) were classroom teachers, 2.3% identified as support staff, and 11.6% identified as “other,” with write-in responses including Special Education teacher and Speech Language Pathologist.

### 3.2. Descriptive Statistics

Participants used a Likert scale (1–10) to indicate the extent to which they use trauma-sensitive behavior management strategies in the classroom. On average, staff most strongly endorsed using direct, brief commands (M = 7.26), followed by active ignoring (M = 6.72), labeled praise (M = 6.35), coping boxes (6.16), and cupcake breathing (4.93). These scores were averaged to produce an index of overall trauma-sensitive classroom skills use (M = 6.30). This index was used in the mediation model. The average participant rated the importance of implementing the principles of a trauma-sensitive school at 8.31 out of 10, rated their confidence in their abilities to implement these principals at a 6.24, and rated their readiness to make a change to implement these principles at a 7.79. These three indicators were averaged to create a readiness to change index, the average of which was 7.44 out of 10. Finally, the average ARTIC score for this sample was 5.09 out of 7. Readiness to change was significantly correlated with trauma-sensitive skill use and ARTIC scores. Skill use was also significantly correlated with ARTIC scores (see Table 1).

### 3.3. Mediation Model

**Figure 1:**
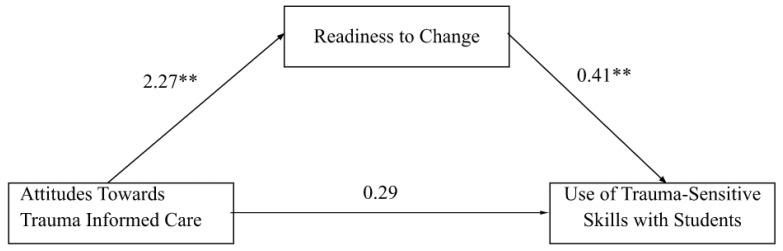
Standardized Regression Coefficients for the Relationship Between Attitudes Toward Trauma-Informed Care and Use of Trauma-Sensitive Skills as Mediated by Readiness to Change. ** *p* < 0.01.

To test the hypothesis that an individual’s readiness to change would mediate the relation between attitudes related to trauma-informed care and trauma-sensitive skill use, a simple mediation analysis was performed using Hayes PROCESS model 4 in SPSS. This model allows for multiple mediators between X and Y, though our model specified only one [25]. The predictor variable for the analysis was ARTIC, the outcome variable was trauma-sensitive skills use, and the mediator variable was readiness to change. As predicted, the relationship between attitudes related to trauma-informed care and use of trauma-sensitive classroom management skills was mediated by readiness to change as indicated by a significant indirect effect of ARTIC on skills use. As Figure 1 illustrates, the standardized regression coefficient between attitudes related to trauma-informed care and readiness to change was statistically significant, as was the standardized regression coefficient between readiness to change and trauma-sensitive skills use. The standardized indirect effect was (β = 0.19, bootstrapped SE = 0.12, 95% LLCI = 0.04, 95% ULCI = 0.49). We tested the significance of this indirect effect using bootstrapping procedures. Unstandardized indirect effects were computed for each of the 5000 bootstrapped samples, and the 95% confidence interval was computed. The bootstrapped unstandardized indirect effect was 0.42 and the 95% confidence interval ranged from 0.08 to 1.13; thus, the indirect effect was statistically significant as the confidence interval did not include zero. The proportion of the total effect of attitudes towards trauma-informed care on use of trauma-sensitive practices that operates indirectly through readiness to change was 58.7%.

## 4. Discussion

Exposure to adverse childhood experiences is associated with negative short-term effects as well as long-term negative mental, cognitive, and physical health outcomes [4,5]. Many symptoms of childhood trauma exposure are impairing in the school setting. These symptoms can interfere with a child’s ability to learn, attend school, and meet behavioral expectations. As such, it is important that school staff feel adequately equipped to recognize and respond to students exhibiting trauma symptoms in the classroom in ways that promote healing and prevent re-victimization. Additionally, schools are uniquely situated to serve as a safe space for children outside of the home. Thus, there has been a national push for schools to adopt trauma-sensitive practices, and many schools across the U.S. have begun developing trauma-sensitive policies and practices. However, the extent to which teachers and school staff are ready to implement trauma-informed practices in the classroom as well as the mechanisms that drive the uptake of these learned skills and practices remains unclear. This study addresses this gap.

Specifically, the purpose of the present study was to examine predictors of educators’ use of trauma-sensitive classroom management practices. The average ARTIC score in this sample was 5.09 out of 7, indicating moderately positive attitudes related to trauma-informed care. As this is a relatively new measure, national norms have not been established. However, similar attitudes have been found in other educator samples. For example, one study of 147 educators across 19 schools reported a mean ARTIC score of 5.07 [26]. Consistent with previous theoretical and empirical literature, we hypothesized that attitudes towards trauma-informed care would predict the use of trauma-sensitive practices. This relationship was obtained; however, the effect size was not large (r = 0.33; medium effect size).

Second, this study extends the existing literature by proposing the novel hypothesis that this relationship would be explained by educators’ readiness to change. Results confirmed that the association between favorable attitudes toward trauma-informed care and the self-reported implementation of evidence-based classroom strategies was fully mediated by participants’ readiness to change. This aligns with previous research which suggests that favorable attitudes toward trauma-informed care are associated with readiness to change and that readiness to change is associated with adoption of trauma-sensitive strategies [17,18]. The present study extends these findings by highlighting the mediating role of readiness to change on the relationship between attitudes toward trauma-informed care and adoption of trauma-sensitive classroom strategies among school staff.

Though schools across the world are eager to provide trauma-sensitive training to their teachers and become trauma-sensitive systems, once the training has been conducted, administrators are often left wondering how to persuade their educators to implement the new trauma-sensitive strategies they just learned. The results from this study indicate that having a trauma lens (i.e., positive attitudes towards trauma-informed care) might be necessary but not sufficient for an educator to adopt the use of trauma-sensitive practices. In the current study, the mean scores for the importance of implementing trauma-informed strategies were high but confidence in implementation was considerably lower. Schools and trauma-sensitive training programs must help educators believe that trauma-sensitive care is important and that they are personally ready to adopt new practices. In addition, it may be particularly important for programs to help educators build confidence in their ability to implement new strategies through tailored training experiences, such as educator coaching. Coaching and experiential training may assist educators who are lacking confidence in implementing their skills.

Limitations of this study include a relatively small sample size of educators, the utilization of self-report scales with a relatively small number of items and lower than optimal internal consistency, and the limited number of trauma-informed strategies which were taught and assessed. Reported use of the various strategies also varied substantially. For example, educators endorsed using brief, direct commands more frequently than cupcake breathing, suggesting that some techniques might be more age-appropriate, easier to implement, or familiar than others. Future studies should also incorporate observations of classroom management strategies to validate teacher self-report, ensure that the strategies are being implemented efficaciously, and to strengthen the conclusions that can be drawn from the current study.

Nonetheless, a strength of the current study includes the context in which it occurred. This study was conducted in a school setting in which the entire staff was able to receive the initial trauma-sensitive schools training, not just a select group of teachers attending an off-site training. Tailored materials were provided to the school and clinicians carrying out the training were known and respected by the school staff, due to their previous work on the APPLE project. Further, due to the school’s location in a high violence, under-resourced neighborhood, it is likely that a considerable number of children in the school had experienced ACEs. Thus, the potential impact of having teachers in this setting adopt greater trauma-sensitive teaching strategies could be transformative for the children in their care. Additionally, all of the trauma-sensitive behavior management techniques that were assessed have been supported in the trauma-informed literature [21,22]. Lastly, consideration of readiness to change as a mediator between attitudes towards trauma-informed care and adoption of classroom strategies was a notable strength, as this relationship had not yet been considered empirically.

Ultimately, this study provides promising evidence that readiness to change may be as critical to implementation as knowledge of trauma-sensitive skills and the embodiment of the trauma lens, given that readiness to change accounted for a significant amount of variance in the relationship between attitude and skill adoption. In fact, in other settings, consultants are assessing organizational readiness to change prior to the adoption of new initiatives and demonstrating that readiness is a function of motivation times general capacity times innovation-specific capacity; results suggest that tailored attention to gaps in readiness can enhance implementation efforts considerably [27]. Continued research into the role of readiness to change, as well as strategies to increase school-level and staff-level readiness to change, is likely to facilitate the uptake of trauma-sensitive practices and policies in the school setting.

## Figures and Tables

**Table 1:** Correlations among scores on the ARTIC, Readiness to Change, and Self-Reported Skill Use.

	Skill Use	Readiness to Change	ARTIC
1. ARTIC	0.33 *	0.44 **	--
2. Readiness to Change	0.49 **	--	--
Mean (SD)	6.30 (SD)	7.44 (1.80)	5.09 (0.73)
Skew	0.61	−0.44	0.07
Range	7.0	7.67	3.0

* *p* < 0.05, ** *p* < 0.01.

## Data Availability

The data presented in this study are available on request from the corresponding author.

## References

[1] Bronfenbrenner U. (1994). Ecological models of human development. Read. Dev. Child..

[2] Felitti V.J., Anda R.F., Nordenberg D., Williamson D.F., Spitz A.M., Edwards V., Marks J.S. (1998). Relationship of childhood abuse and household dysfunction to many of the leading causes of death in adults: The Adverse Childhood Experiences (ACE) Study. Am. J. Prev. Med..

[3] Hughes K., Bellis M.A., Hardcastle K.A., Sethi D., Butchart A., Mikton C., Jones L., Dunne M.P. (2017). The effect of multiple adverse childhood experiences on health: A systematic review and meta-analysis. Lancet Public Health.

[4] Mersky J.P., Topitzes J., Reynolds A.J. (2013). Impacts of adverse childhood experiences on health, mental health, and substance use in early adulthood: A cohort study of an urban, minority sample in the US. Child Abuse Negl..

[5] Plumb J.L., Bush K.A., Kersevich S.E. (2016). Trauma-sensitive schools: An evidence-based approach. Sch. Soc. Work J..

[6] Larson S., Chapman S., Spetz J., Brindis C.D. (2017). Chronic childhood trauma, mental health, academic achievement, and school-based health center mental health services. J. Sch. Health.

[7] Substance Abuse and Mental Health Services Administration (2014). SAMHSA’s Concept of Trauma and Guidance for a Trauma-Informed Approach.

[8] Ko S.J., Ford J.D., Kassam-Adams N., Berkowitz S.J., Wilson C., Wong M., Brymer M.J., Layne C.M. (2008). Creating trauma-informed systems: Child welfare, education, first responders, health care, juvenile justice. Prof. Psychol. Res. Pract..

[9] McIntyre E.M., Baker C.N., Overstreet S. (2019). Evaluating foundational professional development training for trauma-informed approaches in schools. Psychol. Serv..

[10] Brown E.C., Freedle A., Hurless N.L., Miller R.D., Martin C., Paul Z.A. (2020). Preparing teacher candidates for trauma-informed practices. Urban Educ..

[11] Rodger S., Bird R., Hibbert K., Johnson A.M., Specht J., Wathen C.N. (2020). Initial teacher education and trauma and violence informed care in the classroom: Preliminary results from an online teacher education course. Psychol. Sch..

[12] Baker C.N., Brown S.M., Wilcox P.D., Overstreet S., Arora P. (2016). Development and psychometric evaluation of the Attitudes Related to Trauma-Informed Care (ARTIC) scale. Sch. Ment. Health.

[13] Wendel E.L. (2018). Assessing Teacher Attitudes Related to Trauma-Informed Care in Three Urban High Schools. Ph.D. Thesis.

[14] Weiner B.J., Nilsen P., Birken S.A. (2018). A theory of organizational readiness for change. Handbook on Implementation Science.

[15] Resnicow K., DiIorio C., Soet J.E., Ernst D., Borrelli B., Hecht J. (2002). Motivational interviewing in health promotion: It sounds like something is changing. Health Psychol..

[16] LaBrie J., Quinlan T., Schiffman J., Earleywine M. (2005). Performance of alcohol and safer-sex readiness to change rulers compared to readiness to change questionnaires. Psychol. Addict. Behav..

[17] Marvin A.F., Volino Robinson R. (2018). Implementing trauma-informed care at a non-profit human service agency in Alaska: Assessing knowledge, attitudes, and readiness for change. J. Evid. Inf. Soc. Work.

[18] Kirst M., Aery A., Matheson F.I., Stergiopoulos V. (2017). Provider and consumer perceptions of trauma-informed practices and services for substance use and mental health problems. Int. J. Ment. Health Addict..

[19] Winters A.M., Collins-Camargo C., Antle B.F., Verbist A.N. (2020). Implementation of system-wide change in child welfare and behavioral health: The role of capacity, collaboration, and readiness for change. Child. Youth Serv. Rev..

[20] Alabama State Department of Education Fall Free Lunch 2017–2018. https://www.alsde.edu/dept/data/Pages/freelunch-all.aspx..

[21] Brunzell T., Stokes H., Waters L. (2016). Trauma-informed flexible learning: Classrooms that strengthen regulatory abilities. Int. J. Child Youth Fam. Stud..

[22] Cavanaugh B. (2016). Trauma-informed classrooms and schools. Beyond Behav..

[23] Baker C.N., Brown S.M., Overstreet S., Wilcox P.D. (2020). Validation of the Attitudes Related to Trauma-Informed Care Scale (ARTIC). Psychol. Trauma Theory Res. Pract. Policy.

[24] DiClemente C.C., Velasquez M.M., Miller W.R., Rollnick S. (2002). Motivational interviewing and the stages of change. Motivational Interviewing: Preparing People for Change.

[25] Hayes A.F. (2017). Introduction to Mediation, Moderation, and Conditional Process Analysis: A Regression-Based Approach.

[26] Grybush A.L. (2020). Exploring Attitudes Related to Trauma-Informed Care among Teachers in Rural Title I Elementary Schools: Implications for Counselors and Counselor Educators. Ph.D. Thesis.

[27] Scott V.C., Kenworthy T., Godly-Reynolds E., Bastien G., Scaccia J., McMickens C., Rachel S., Cooper S., Wrenn G., Wandersman A. (2017). The Readiness for Integrated Care Questionnaire (RICQ): An instrument to assess readiness to integrate behavioral health and primary care. Am. J. Orthopsychiatry.

